# Chemical Space Exploration of Oxetanes

**DOI:** 10.3390/ijms21218199

**Published:** 2020-11-02

**Authors:** Fernando Rodrigues de Sá Alves, Rafael M. Couñago, Stefan Laufer

**Affiliations:** 1 Department of Pharmaceutical and Medicinal Chemistry, Institute of Pharmaceutical Sciences, Faculty of Science, Eberhard Karls University of Tübingen, 72074 Tübingen, Germany; fernandorodriguessalves@gmail.com; 2 Centro de Química Medicinal (CQMED), Centro de Biologia Molecular e Engenharia Genética (CBMEG), Structural Genomics Consortium, Departamento de Genética e Evolução, Instituto de Biologia Universidade Estadual de Campinas (UNICAMP), Campinas 13083-875, SP, Brazil; rafaelcounago@gmail.com; 3 Tübingen Center for Academic Drug Discovery, 72076 Tübingen, Germany

**Keywords:** oxetane, chemical space, nonclassical isosterism, Buchwald–Hartwig reaction, kinases

## Abstract

This paper focuses on new derivatives bearing an oxetane group to extend accessible chemical space for further identification of kinase inhibitors. The ability to modulate kinase activity represents an important therapeutic strategy for the treatment of human illnesses. Known as a nonclassical isoster of the carbonyl group, due to its high polarity and great ability to function as an acceptor of hydrogen bond, oxetane seems to be an attractive and underexplored structural motif in medicinal chemistry.

## 1. Introduction

Oxetane is a four-membered ring having an oxygen atom with an intrinsic ring strain of 106 kJ.mol^−1^, which adopts a planar structure with a puckering angle of only 8.7° at 140 K (10.7° at 90 K). The addition of substituents into the oxetane ring can increment unfavorable eclipsing interactions, resulting in a more puckered conformation [1].

The strained C−O−C bond angle exposes the oxygen lone pair of electrons, allowing the oxetane to act as a good hydrogen-bond acceptor as well as donating electron density as a Lewis base. It was also observed experimentally that oxetanes form more effective H-bonds than other cyclic ethers [2]. Similarly, oxetanes compete as H-bond acceptors with the majority of carbonyl functional groups like aliphatic ketones, aldehydes, and esters [3]. These structural features are important for many of the beneficial properties of substituted oxetanes.

**Figure 1:**
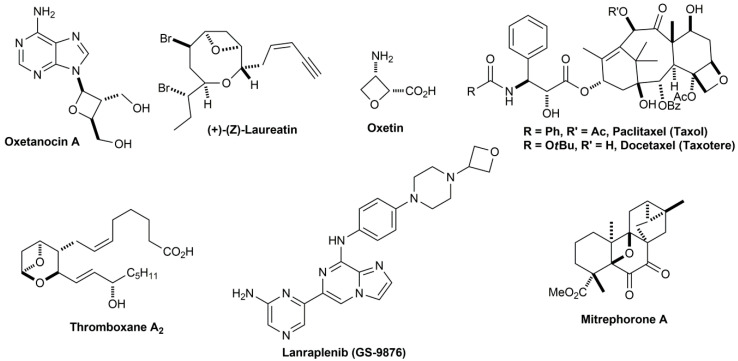
Natural products and marketed drugs containing an oxetane group.

Some examples of compounds bearing the oxetane are oxetanocin A, thromboxane A_2_, mitrephorone A, oxetine and laureatin, as well as the marketed chemotherapy drugs Taxol and Taxotere and a new kinase inhibitor GS-9876 (Figure 1).

**Figure 2:**
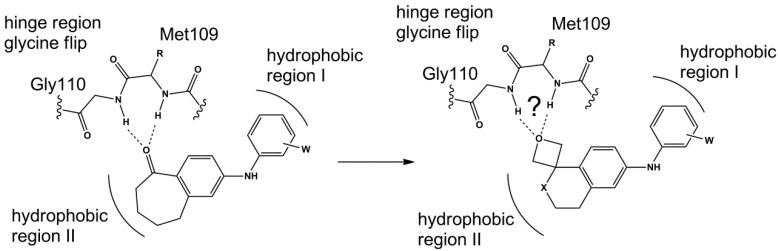
Schematic of the interactions of benzosuberone and oxetane analogs in the hinge region.

In medicinal chemistry, an oxetane group is employed not only to change the conformational preference of the main scaffold but also to influence physicochemical and biochemical properties such as lipophilicity, aqueous solubility, and metabolic stability [4]. Carreira and co-workers from ETH Zurich have been using oxetanes as surrogates of a carbonyl group in order to generate building blocks providing new opportunities of molecular diversity [5,6,7]. The group of Professor Bull from Imperial College London has also been working extensively on the synthesis and new methodologies for obtaining compounds bearing an oxetane [8,9,10]. Recently, Blomgren and co-workers have reported the discovery of a second-generation spleen tyrosine kinase inhibitor, GS-9876 (lanraplenib), which has human pharmacokinetic properties suitable for once-daily administration and it is used against autoimmune diseases [11]. Known as a nonclassical isoster of the carbonyl group [12], due to its high polarity and great ability to function as an acceptor of hydrogen bond, the oxetane still remains an underexplored structural motif in drug discovery, especially in the kinase field, in which the oxetane could bind the “hinge” region (Figure 2). Nevertheless, the conformational variations or the directional hydrogen bonding differences between sp_2_- and sp_3_-hybridized oxygen atoms could be a benefit or a drawback for that type of interaction.

## 2. Results and Discussion

**Figure 3:**
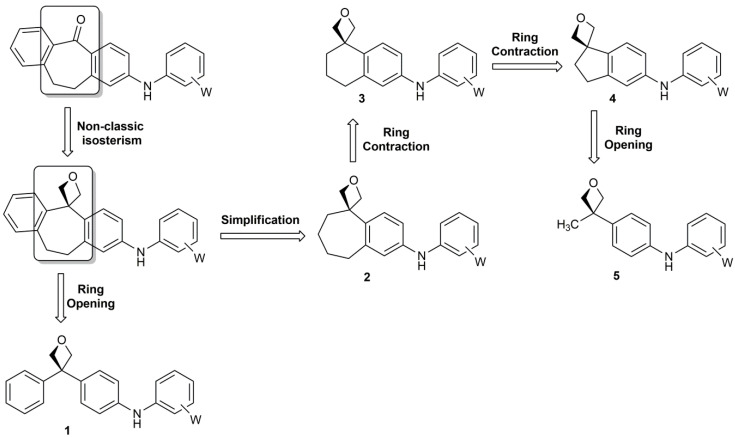
Design of new oxetane derivatives.

Our main goal was to synthesize new oxetane derivatives to further extend the therapeutical kinase space. We proposed the nonclassical isosteric replacement of the carbonyl group of some kinase inhibitors bearing dibenzosuberone and benzophenone scaffolds by an oxetane moiety (Figure 3). This substitution could lead to an interaction of the oxetane group with the “hinge” region. All protein kinases contain a sequence of amino acids comprising the “hinge” between the two lobes of the catalytic domain. The backbone atoms of the hinge region contain the critical hydrogen bond donor and acceptor atoms anchoring ATP-binding and allowing phosphorylation of the substrate [13].

**Figure 4:**
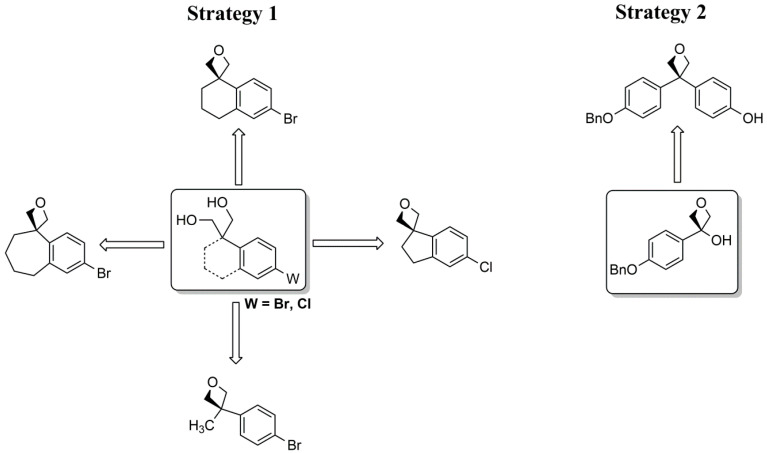
Strategies used to synthesize the oxetane scaffolds.

3,3-disubstituted oxetanes can be readily synthesized from 1,3-diols by conversion of the latter into a halohydrin (or another good leaving group) and then by addition of a strong base to close the ring through an intramolecular Williamson etherification reaction [14,15]. However, in the case of 1,3-diols bearing two aryl groups, an 1,4-elimination process namely Grob fragmentation occurs instead of a cyclization, leading to an alkene [16,17,18]. Thus, a different approach through a catalytic functionalization of tertiary alcohols was used in order to obtain 3,3-diphenyloxetane derivatives (Figure 4).

**Scheme 1:**
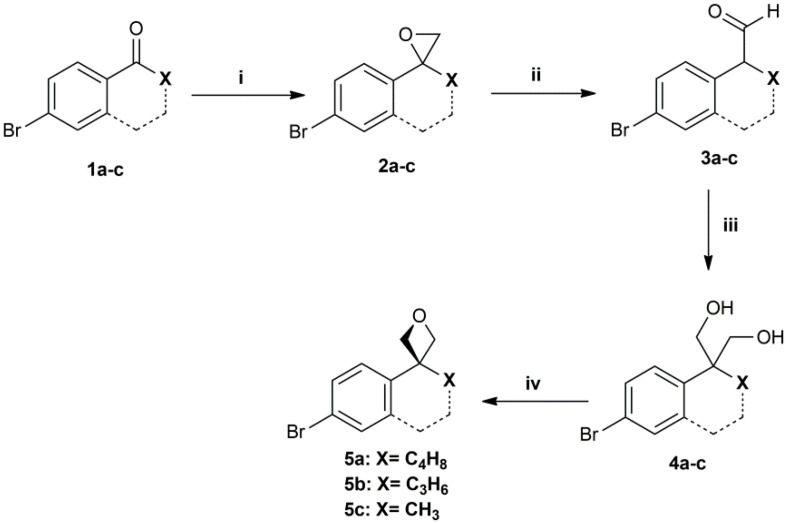
Reagents and Conditions: (i) (CH_3_)_3_SI, NaH, DMSO, r.t. (ii) ZnI_2_, benzene, reflux. (iii) CH_2_O, KOH, ethylene glycol, reflux. (iv) 1. TDAP, CCl_4_, THF, −40 °C; 2. NaOMe, MeOH, reflux.

The ketones **1a–c** reacted with dimethylsulfonium methylide in a Corey–Chaykovsky epoxidation reaction [19], leading to the epoxide derivatives **2a–c**. In the next step, the aldehydes **3a–c** were obtained through a Lewis acid catalyzed epoxide rearrangement by a 1,2-hydride shift, namely Meinwald rearrangement [20]. Then a crossed Cannizzaro reaction was performed [21], reacting the aldehydes with formaldehyde in concentrated basic conditions, obtaining the 1,3-diol derivatives **4a–c**. For the synthesis of the oxetanes applying strategy 1, we used the concept of hydroxyl replacement via oxyphosphonium salts, in which phosphonium salts can also be formed by reaction of different trivalent phosphorus compounds with oxidizing electrophiles, like the tris(dimethylamino)phosphine-carbon tetrachloride component system. The produced phosphonium intermediates can be trapped with an alcohol to form alkoxyphosphonium salts, which can then undergo nucleophilic displacement [22]. Based on the works of Castro and Selve [23], the dialkyloxetanes **5a–c** were obtained in good yields (Scheme 1).

**Scheme 2:**
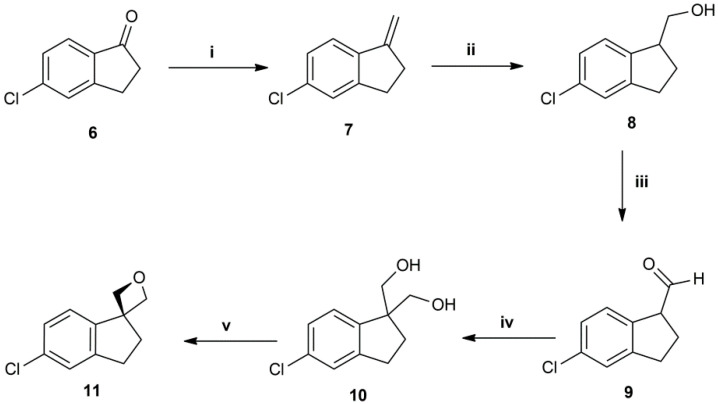
Reagents and Conditions: (i) 1. Ph_3_PMeI, *t*-BuOK, THF, 0 °C; 2. 5-chloro-indanone, 0 °C to r.t. (ii) 1. BH_3_, THF, 0 °C; 2. NaOH, H_2_O_2_, 0 °C—r.t. (iii) Dess–Martin periodinane, CH_2_Cl_2_, r.t. (iv) CH_2_O, KOH, ethylene glycol, reflux. (v) 1. TDAP, CCl_4_, THF, −40 °C; 2. NaOMe, MeOH, reflux.

In the case of (5-chloro-2,3-dihydro-1H-indene-1,1-diyl)dimethanol, as the Corey–Chaykovsky epoxidation reaction did not work, the 5-chloro-indanone **6** was converted in compound **7** through a Wittig reaction [24]. Then a Brown hydroboration reaction was carried out followed by oxidation of the borate, in which the alkene was converted into the primary alcohol **8**, using hydrogen peroxide in basic conditions [25,26]. The alcohol **8** was then oxidized to the aldehyde **9** using Dess–Martin periodinane [27] and subsequently converted into the diol **10** and oxetane **11** employing the same reactions mentioned before (Scheme 2).

**Scheme 3:**
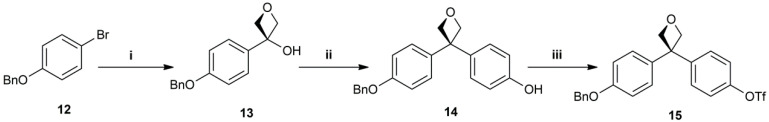
Reagents and Conditions: (i) *n*-BuLi, oxetan-3-one, THF, −78 °C. (ii) Li(NTf_2_), Bu_4_NPF_6_, CHCl_3_, 40 °C. (iii) triflic anhydride, pyridine, CH_2_Cl_2_, r.t.

The 3,3-diaryloxetane scaffold **14** was synthesized based on the works of Croft and co-workers [28] through the catalytic functionalization of a tertiary alcohol to construct a fully substituted carbon center, using Li as a catalyst in a Friedel–Crafts alkylation reaction [29]. A halogen-metal exchange reaction of BuLi and compound **12**, followed by a nucleophilic addition of the organolithium to the carbonyl group of the oxetan-3-one, gave rise to compound **13**. The ^1^H NMR spectrum shows two doublets related to the methylene groups of oxetane, at 4.88 and 4.91 ppm. Additionally, the OH group could be detected as a broad singlet at 2.92 ppm. Afterwards, the tertiary alcohol **13** was reacted with phenol, leading to the diaryloxetane **14** in a 60% yield. Compound **14** was then converted into the triflate derivative **15** in a 62% yield [30] (Scheme 3).

For the last step, the optimal experimental conditions were established for the Buchwald–Hartwig reaction of the halide scaffolds with arylamines, using a catalyst system composed of Pd(OAc)_2_, XPhos and Cs_2_CO_3_, in a methodology adapted from our research group for dibenzosuberone derivatives [31]. For the coupling of the aryl triflate with arylamines, a modified method from Buchwald’s group was employed [32,33], through a catalyst system composed of Pd(OAc)_2_, BINAP and Cs_2_CO_3_. No formation of phenol was detected. The deprotection of the benzyloxyl group was carried out with Pd/C under H_2_ atmosphere at r.t. in a mixture of ethanol/acetonitrile, leading to the final compounds in quantitative yields. The oxetane derivatives were reacted with different arylamines. The 2,4-difluoroaniline was initially selected, based on previous works of our laboratory, where it had demonstrated good potency for some kinase inhibitors [34]. Then other modifications were carried out in the arylamine subunit, selecting both electron-withdrawing and donating groups. Additionally, aniline was chosen as a non-substituted group to complete the design of new derivatives (Table 1).

**Figure 5:**
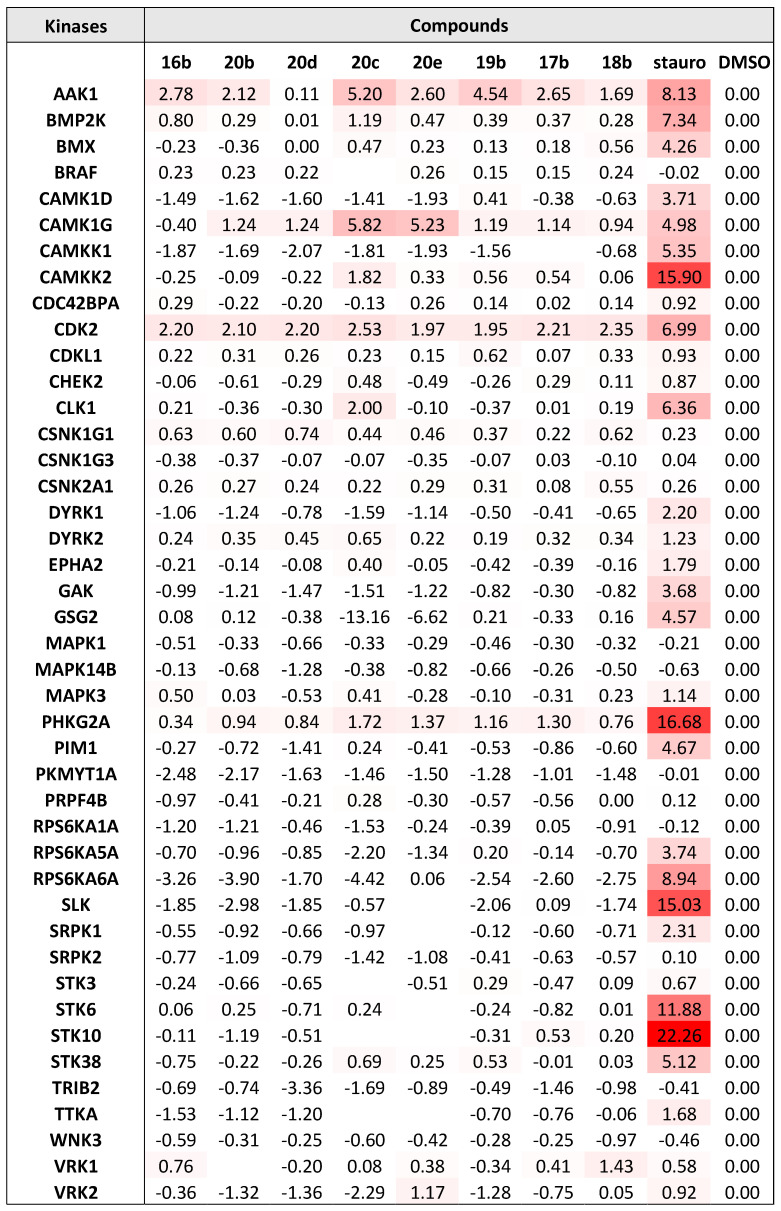
Results obtained from a kinase panel using differential scanning fluorimetry (DSF).

In order to identify novel scaffolds for molecular recognition in kinases, we selected some compounds to be screened in a panel of kinases using the differential scanning fluorimetry (DSF) method [35,36] at SGC-Unicamp. This technique allows selectivity profiling of any protein kinase without prior knowledge of either substrate or activity of the kinase under investigation. The most promising results were obtained for the CAMK1G calcium/calmodulin dependent protein kinase I gamma and for AAK1 AP2-associated protein kinase 1, as depicted in Figure 5, showing Tm shifts greater than 2 °C. The pankinase inhibitor staurosporine (stauro) served as a positive control in the assay.

**Figure 6:**
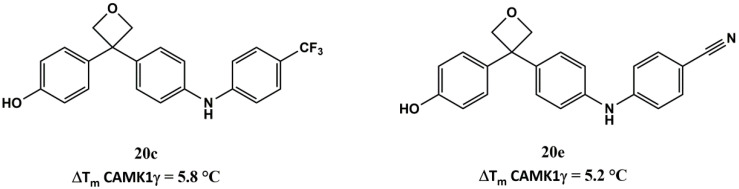
∆Tm values obtained for the CAMK1G calcium/calmodulin dependent protein kinase I gamma.

4-(3-phenyloxetan-3-yl)phenol derivatives **20c** and **20e** showed a ΔTm of 5.8 °C and 5.2 °C at 10 µM, respectively against CAMK1G (Figure 6). These results suggest that electron-withdrawing groups on the aryl ring are well tolerated, in which the 4-CF_3_ and 4-CN analogs showed similar values. However, both an electron-donating group like 4-OCH_3_ (**20d**) and the group 2,4-difluoryl (**20b**) were detrimental to CAMK1G interaction. Calcium–calmodulin dependent protein kinase I (CaMKI) is part of a calmodulin-dependent protein kinase cascade and is implicated in various physiological processes, being expressed in many tissues. CaMKI regulates transcriptional activator activity, the cell cycle, cell differentiation, actin filament organization and neurite outgrowth [37]. However, the role of CAMK1G is still mainly unclear.

**Figure 7:**
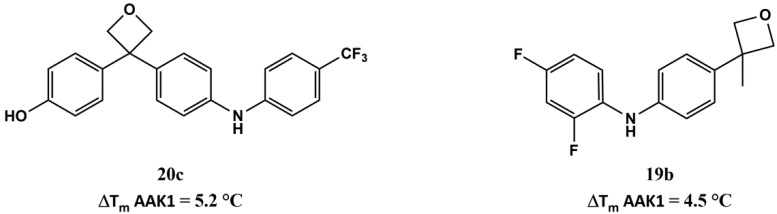
∆Tm values obtained for the AAK1 AP2-associated protein kinase 1.

Compounds **20c** and **19b** showed a ΔTm of 5.2 °C and 4.5 °C, respectively against AAK1 (Figure 7). Adaptor-associated kinase 1 (AAK1), a member of Ark1/Prk1 family of serine/threonine kinases, is involved in the endocytic pathway. Although AAK1 has been mostly related to the regulation of receptor endocytosis, several studies suggested that AAK1 may be associated to psychiatric and neurological disorders [38]. Recently, Kostich and co-workers have showed that the inhibition of AAK1 kinase could be a novel therapeutic approach for the treatment of neuropathic pain [39]. The inhibition of AAK1 also showed to be important as an antivirus strategy, especially against HCV [40,41]. In addition to these preliminary results, new structural modifications and experiments to measure the K_d_ and IC_50_ will be carried out for a better understanding of the structure-activity relationship.

## 3. Materials and Methods

All commercially available reagents and solvents were used without further purification. ^1^H and ^13^C nuclear magnetic resonance (NMR) spectra were determined in DMSO-d_6_, CDCl_3_ and Acetone-d_6_ solutions using a Bruker Avance 200 (^1^H = 200 MHz; ^13^C = 50 MHz) or a Bruker Avance 400 (^1^H = 400 MHz; ^13^C = 100 MHz) spectrometer. The chemical shifts are given in parts per million (ppm) from solvent residual peaks and the coupling constant values (*J*) are given in Hz. Signal multiplicities are represented by: s (singlet), d (doublet), dd (double doublet), t (triplet), m (multiplet) and br (broad signal). The evaluation of spectra was performed using the MestReNova program. Infrared spectra were obtained using a Thermo Scientific Nicolet’s Avatar iS10 spectrometer equipped with a smart endurance diamond ATR unit for direct measurements. ESI mass spectra were obtained from a TLC-MS interface CAMAG in negative mode [M − H^+^]. EI-MS mass spectra were obtained from the Mass Spectrometry Department of the Institute of Organic Chemistry, University of Tübingen: Finnigan MAT, TSQ 70 Tripel quadrupol; ion source temperature at 200 °C; temperature of evaporation: 30–300 °C; ionization energy: 70 eV. The compounds were purified through column chromatography, using silica gel as stationary phase (Geduran^®^ Si 60, 0.063–0.200 mm). The purity of compounds was determined by HPLC (Merck Hitachi L-6200 intelligent pump, Merck Hitachi AS-2000 auto sampler, Merck Hitachi L-4250 UV vis detector) using a ZORBAX Eclipse XDB C8 column (5 mm), employing a gradient of 0.01 M KH_2_PO_4_ (pH 2.3) and methanol as solvent system with a flow rate of 1.5 mL/min and detection at 254 nm.

Kinase selectivity assay: Starting from 100 µM protein stocks, all the kinases present in the panel were diluted to 1 µM in buffer 100 mM K_2_HPO_4_ pH 7.5 containing 150 mM NaCl, 10% glycerol and 1X dye (Applied Biosystems catalog 4461806). The protein/dye mixture was transferred column-wise to a 384-well PCR microplate with 20 µL per well. Compounds at 10 mM concentration in DMSO (from a separate plate arranged in rows) were added next, in 20 nL volume, using a liquid handling device setup with a pin head to make a final 10 µM compound in the assay plate. The final DMSO concentration in all wells is 0.2%, including the reference with DMSO only. Thermal shift data was measured in a qPCR instrument (Applied Biosystems QuantStudio 6) programmed to equilibrate the plate at 25 °C for 5 min followed by ramping the temperature to 95 °C at a rate of 0.05 °C per second. Data was processed on Protein Thermal shift software (Applied Biosystems) fitting experimental curves to a Boltzmann function to calculate differential thermal shifts (dTm) referenced to protein/dye in 0.2% DMSO. The kinases were cloned, expressed (bacteria or insect cell expression) and purified at the laboratory of SGC.

### 3.1. General Procedure A—Corey-Chaykovsky Epoxidation

4.0 eq. of NaH (60% dispersion in mineral oil) was placed in a three-necked flask under argon and then DMSO (0.6 M) and the ketone were added, after which 2.25 eq. of trimethylsulfonium iodide was also added, all at once. The reaction mixture was stirred at ambient temperature. After controlling through TLC, the mixture was poured over ice and extracted with diethyl ether and ethyl acetate (1:1). The extracts were combined, washed with brine and afterwards dried over Na_2_SO_4_. The solvent was removed under vacuum, and the residue was used without purification for the next step. Adapted from Winter [19].

*2-bromo-6,7,8,9-tetrahydrospiro[benzo[7]annulene-5,2′-oxirane]* (**2a**): Yellow oil. 95% yield. ^1^H-NMR (200 MHz, CDCl_3_) δ in ppm: 1.62–1.80 (m, 2H), 1.80–2.15 (m, 2H), 2.74 (d, 1H, *J* = 6.0 Hz), 2.82–2.87 (m, 2H), 2.98 (d, 1H, *J* = 6.0 Hz), 7.30 (m, 3H); ^13^C-NMR (50 MHz, CDCl_3_) δ in ppm: 27.0, 28.6, 35.9, 36.0, 53.9, 60.8, 121.2, 126.9, 129.1, 131.8, 140.1, 143.3.

*6-bromo-3,4-dihydro-2H-spiro[naphthalene-1,2′-oxirane]* (**2b**): Yellow solid. 90% yield. ^1^H-NMR (200 MHz, CDCl_3_) δ in ppm: 1.75–1.85 (m, 2H), 2.06–2.11 (m, 2H), 2.83–2.87 (m, 2H), 2.97 (s, 2H), 6.92–6.96 (d, 2H), 7.27–7.29 (s, 1H); ^13^C-NMR (50 MHz, CDCl_3_) δ in ppm: 21.9, 29.6, 31.7, 56.4, 58.9, 121.5, 125.4, 129.6, 131.4, 134.9, 141.7.

*2-(4-bromophenyl)-2-methyloxirane* (**2c**): Yellow solid. 90% yield. ^1^H-NMR (200 MHz, CDCl_3_) δ in ppm: 1.70 (s, 3H), 2.74–2.77 (d, 1H), 2.97–2.99 (d, 1H), 7.22–7.26 (d, *J* = 8.0 Hz, 2H), 7.44–7.48 (d, *J* = 8.0 Hz, 2H); ^13^C-NMR (50 MHz, CDCl_3_) δ in ppm: 21.7, 56.5, 57.1, 121.6, 127.2, 131.6, 140.4.

### 3.2. General Procedure B—Meinwald Rearrangement

The crude epoxide was dissolved in benzene (0.3 M) and 0.3 eq. of ZnI_2_ was added; the reaction mixture was heated at reflux under argon. After controlling through TLC, it was then diluted with ethyl acetate and washed with water. The organic layer was dried with Na_2_SO_4_, and the solvent was evaporated to afford the pure aldehyde, which was used without purification for the next step. Adapted from Snyder et al. [20].

*2-bromo-6,7,8,9-tetrahydro-5H-benzo[7]annulene-5-carbaldehyde* (**3a**): Brown oil. 98% yield. ^1^H-NMR (200 MHz, CDCl_3_) δ in ppm: 1.59–2.25 (m, 6H, CH_2_), 2.68 (t, 2H, CH_2_), 3.71 (d, 1H, CH), 6.95 (d, 1H, *J* = 8.0 Hz), 7.31 (m, 2H), 9.89 (s, 1H).

*6-bromo-1,2,3,4-tetrahydronaphthalene-1-carbaldehyde* (**3b**): Brown oil. 95% yield. ^1^H-NMR (200 MHz, CDCl_3_) δ in ppm: 1.92 (m, 2H), 2.24 (m, 2H), 2.72–2.78 (t, 2H), 3.58 (t, 1H), 6.99–7.03 (d, 1H), 7.30–7.34 (m, 2H), 9.64 (s, 1H); ^13^C-NMR (50 MHz, CDCl_3_) δ in ppm: 20.2, 22.9, 29.1, 51.3, 121.2, 129.4, 129.9, 131.3, 132.6, 140.3, 201.4.

*2-(4-bromophenyl)propanal* (**3c**): Brown oil. 98% yield. ^1^H-NMR (200 MHz, CDCl_3_) δ in ppm: 1.41–1.45 (d, 3H), 3.55–3.66 (q, 1H), 7.06–7.11 (d, 2H), 7.48–7.52 (d, 2H), 9.65 (s, 1H); ^13^C-NMR (50 MHz, CDCl_3_) δ in ppm: 14.7, 52.5, 121.7, 130.1, 132.3, 136.8, 200.5.

### 3.3. General Procedure C—Crossed Cannizzaro Reaction

A mixture of 35% formalin, KOH 50% aqueous solution and the aldehyde in ethylene glycol (0.4 M) was heated at reflux overnight. Water was added to the resulting mixture, and the organic layer was extracted with CH_2_Cl_2_, washed with brine, and dried over Na_2_SO_4_. After concentration of the solution, hexane was added immediately with vigorous stirring, and then the mixture was cooled to 0 °C. White precipitate was collected, washed with hexane, and dried under air to obtain the diol. Adapted from Sato et al. [21].

*(2-bromo-6,7,8,9-tetrahydro-5H-benzo[7]annulene-5,5-diyl)dimethanol* (**4a**): White solid. 61% yield. HPLC: 6.948 min (83%) at 254 nm. IR (ATR): 3276, 2931, 2882, 2855, 1556, 1445 cm^−1^. ESI-MS: Calculated: 284.0 for C_13_H_17_BrO_2_. Found: 283 [M − H]^−^. ^1^H-NMR (200 MHz, CDCl_3_) δ in ppm: 1.26 (s, 2H, CH_2_), 1.79 (m, 4H, CH_2_), 2.31 (s, 2H, CH_2_), 2.90 (br, 2H), 3.88 (d, 2H, *J* = 10 Hz), 4.04 (d, 2H, *J* = 12 Hz), 7.27–7.30 (m, 3H); ^13^C-NMR (50 MHz, CDCl_3_) δ in ppm: 23.9, 27.3, 30.9, 35.9, 48.6, 68.5, 120.7, 129.3, 129.9, 134.4, 140.2, 144.8.

*(6-bromo-1,2,3,4-tetrahydronaphthalene-1,1-diyl)dimethanol* (**4b**): White solid. 45% yield. HPLC: 6.950 min (96%). IR (ATR): 3269, 2928, 2868, 2360, 1479 cm^−1^. ESI-MS: Calculated: 270.03 for C_12_H_15_BrO_2_. Found: 269 [M − H]^−^. ^1^H-NMR (200 MHz, CDCl_3_) δ in ppm: 1.77–1.92 (m, 4H), 1.93 (s, 2H), 2.72–2.78 (t, 2H), 3.71–3.76 (d, 2H, *J* = 10 Hz), 3.87–3.92 (d, 2H, *J* = 10 Hz), 7.27 (s, 3H); ^13^C-NMR (50 MHz, CDCl_3_) δ in ppm: 18.9, 27.7, 30.5, 43.3, 69.7, 120.6, 128.9, 129.2, 132.4, 136.9, 141.2.

*(5-chloro-2,3-dihydro-1H-indene-1,1-diyl)dimethanol* (**10**): White solid. 30% yield. HPLC: 5.580 min (98%). IR (ATR): 3280, 2933, 2877, 2843, 1451, 1200, 824 cm^−1^. ESI-MS: Calculated: 212.0 for C_11_H_13_ClO_2_. Found: 211 [M − H]^−^. ^1^H-NMR (200 MHz, CDCl_3_) δ in ppm: 2.04–2.12 (m, 4H), 2.88–2.96 (t, 2H), 3.72–3.87 (q, 4H), 7.12–7.25 (m, 3H); ^13^C-NMR (50 MHz, CDCl_3_) δ in ppm: 30.2, 31.4, 54.2, 68.2, 125.2, 125.4, 126.8, 133.6, 143.2, 146.8.

*2-(4-bromophenyl)-2-methylpropane-1,3-diol* (**4c**): White solid. 67% yield. HPLC: 5.280 min (100%). ESI-MS: Calculated: 244.01/246.01 for C_10_H_13_BrO_2_. Found: 243/245 [M − H]^−^. ^1^H-NMR (200 MHz, CDCl_3_) δ in ppm: 1.25 (s, 3H), 2.22 (s, 2H), 3.78–3.83 (d, 2H), 3.91–3.97 (d, 2H), 7.29–7.33 (d, 2H), 7.46–7.51 (d, 2H); ^13^C-NMR (50 MHz, CDCl_3_) δ in ppm: 20.9, 44.5, 70.0, 120.8, 128.7, 131.8, 142.3.

### 3.4. General Procedure D—Wittig Reaction

Methyltriphenylphosphonium bromide (1.287 g, 3.601 mmol) was suspended in 15 mL of dry THF at 0 °C. KO*t*Bu (0.404 g, 3.601 mmol) was added to the suspension and a bright yellow color was observed. The mixture was stirred at 0 °C for 30 min. Then 5-chloro-2,3-dihydro-1H-inden-1-one (0.200 g, 1.200 mmol) was added neat and the reaction mixture was stirred at 0 °C to r.t. for 4 h (the solution turned green and then dark brown). Water was added to the mixture and the product was extracted with CH_2_Cl_2_ and dried with Na_2_SO_4_, filtrated and concentrated in vacuum. The residue was purified by column chromatography using *n*-hexane. Adapted from Liwosz and Chemler [24].

*5-chloro-1-methylene-2,3-dihydro-1H-indene* (**7**): Incolor oil. 83% yield. HPLC: 9.720 min (99.4%). ^1^H-NMR (200 MHz, CDCl_3_) δ in ppm: 2.77–2.85 (m, 2H), 2.93–3.00 (m, 2H), 5.04–5.07 (t, 1H), 5.42–5.44 (t, 1H), 7.15–7.19 (d, *J* = 8.0 Hz, 1H), 7.23 (s, 1H), 7.38–7.42 (d, *J* = 8.0 Hz, 1H); ^13^C-NMR (50 MHz, CDCl_3_) δ in ppm: 13.1, 37.6, 119.7, 124.1, 126.3, 129.2, 130.7, 139.5, 144.8, 146.1.

### 3.5. General Procedure E—Brown Hydroboration—Oxidation

A solution of 1.6 g (9.537 mmol) of 5-chloro-1-methylene-2,3-dihydro-1H-indene in 10 mL of THF was treated with a solution of 19.0 mL of BH_3_-THF complex at ice-bath temperature. The reaction mixture was stirred until disappearance of the alkene. It was then cooled in a water-ice bath, and carefully treated with methanol followed by addition of 31.8 mL (95.365 mmol) of NaOH 3.0 M and 25.3 mL (247.959 mmol) of 30% H_2_O_2_. The resulting mixture was stirred at room temperature, poured into water, acidified with 10% HCl, and extracted with ether. The organic layer was dried with Na_2_SO_4_ and the solvent was evaporated to afford the pure alcohol after purification by column in silica gel (20% EtOAc:*n*-hexane). Adapted from Zimmerman and Nesterov [26].

*(5-chloro-2,3-dihydro-1H-inden-1-yl)methanol* (**8**): Yellow oil. 80% yield. HPLC: 9.720 min (99.4%). IR (ATR): 3323, 2940, 2866, 1598, 1471, 1067, 870 cm^−1^. ^1^H-NMR (400 MHz, CDCl_3_) δ in ppm: 1.62 (br s, 1H), 1.91–1.98 (sextet, 1H), 2.23–2.32 (sextet, 1H), 2.82–2.99 (m, 2H), 3.27–3.34 (quintet, 1H), 3.76–3.77 (d, 2H), 7.13–7.15 (d, 1H), 7.19–7.21 (m, 2H); ^13^C-NMR (50 MHz, CDCl_3_) δ in ppm: 28.7, 31.4, 47.1, 65.9, 125.1, 125.3, 126.5, 132.8, 142.5, 146.8.

### 3.6. General Procedure F—Dess–Martin Reaction

To a solution of 0.470 g (2.573 mmol) of (5-chloro-2,3-dihydro-1H-inden-1-yl)methanol in 20 mL of CH_2_Cl_2_ (0.13 M) 1.6 g (3.860 mmol) of Dess–Martin periodate was added. The reaction was allowed to stir at room temperature for 3 h. TLC and HPLC analysis showed formation of the desired product. The solution was diluted with CH_2_Cl_2_ and a 10% Na_2_S_2_O_3_ solution to consume the excess Dess–Martin reagent was added. The mixture was stirred until the two layers separated. The CH_2_Cl_2_ layer was collected, washed with saturated Na_2_CO_3_ solution and dried with Na_2_SO_4_, filtered and evaporated to give the crude aldehyde, which was used in the next step without purification. Adapted from Thongsornkleeb and Danheiser [27].

*5-chloro-2,3-dihydro-1H-indene-1-carbaldehyde* (**9**): Yellow oil. 98% yield. ^1^H-NMR (200 MHz, CDCl_3_) δ in ppm: 2.36–2.45 (m, 2H), 2.95–3.03 (t, 2H), 3.88–3.95 (t, 1H), 7.20 (s, 3H), 9.64 (s, 1H); ^13^C-NMR (50 MHz, CDCl_3_) δ in ppm: 25.8, 31.7, 57.3, 125.5, 126.0, 127.1, 134.0, 137.0, 146.8, 200.2.

### 3.7. General Procedure G—Nucleophilic Addition to the Carbonyl Group

Compound **9** was synthesized according to General Procedure H: 2,6 mL (6.513 mmol) of *n*-BuLi was added dropwise over 5 min to a solution of 1.71 g (6.513 mmol) of 1-(benzyloxy)-4-bromobenzene in 20 mL of THF at −78 °C. The reaction mixture was stirred at −78 °C for a further 30 min. Then, 0.36 g (5.010 mmol) of oxetan-3-one was added dropwise to the reaction mixture. After a further 45 min at −78 °C the reaction mixture was warmed to room temperature then quenched with water. The layers were separated and the aqueous portion extracted with diethylether. The organic extracts were combined, washed with brine, dried over Na_2_SO_4_, filtered and concentrated in vacuum. Purification by column chromatography (40% EtOAc:*n*-hexane) afforded the product. Adapted from Croft et al. [28].

*3-(4-(benzyloxy)phenyl)oxetan-3-ol* (**13**): White solid. 70% yield. HPLC: 6.898 min (98%). ^1^H-NMR (400 MHz, CDCl_3_) δ in ppm: 2.92 (br, 1H), 4.86–4.91 (m, 4H), 5.09 (s, 2H), 7.00–7.02 (d, 2H, *J* = 8.0 Hz), 7.33–7.35 (d, 2H, *J* = 8.0 Hz), 7.38–7.45 (m, 4H), 7.47–7.49 (d,2H, *J* = 8.0 Hz); ^13^C-NMR (100 MHz, CDCl_3_) δ in ppm: 70.2, 75.8, 85.7, 115.2, 126.1, 127.6, 128.2, 128.8, 134.9, 136.9, 158.6.

### 3.8. General Procedure H—Oxetane Formation via Williamson Etherification

The diol was dissolved in THF (0.1 M) and then 4 eq. of CCl_4_ was added. The solution was cooled to −48 °C and then 1.1 eq. of Tris(dimethylamino)phosphine in 1 mL of THF was added slowly to this solution during 5 min. The solution turned slightly turbid. The flask was transferred to an ice-bath and then agitated until it reached room temperature. After controlling using HPLC, the solvent was evaporated and then methanol was added followed by 4 eq. of a 5.4 M solution of sodium methoxide. The mixture was refluxed at 90 °C during 1 h. The reaction was then diluted with ethyl acetate and washed with water. The organic layer was dried with Na_2_SO_4_ and the solvent was evaporated to afford a product after purification by column in silica gel (5% EtOAc: *n*-hexane). Adapted from Castro and Selve [23].

*2-bromo-6,7,8,9-tetrahydrospiro[benzo[7]annulene-5,3′-oxetane]* (**5a**): Yellow oil. 47% yield. HPLC: 8.497 min (93%). EI-MS: Calculated 266.03 for C_13_H_15_BrO. Found: 235.9 [M-CH_2_O]^+●^. ^1^H-NMR (200 MHz, CDCl_3_) δ in ppm: 1.53–1.59 (t, 2H), 1.99–2.01 (t, 2H), 2.12–2.15 (d, 2H), 2.4–2.45 (d, 2H), 4.70–4.73 (d, *J* = 6.0 Hz, 2H), 4.97–5.00 (d, *J* = 6.0 Hz, 2H), 6.92–6.96 (d, 1H), 7.21–7.33 (m, 2H). ^13^C-NMR (50 MHz, CDCl_3_) δ in ppm: 26.4, 26.9, 34.9, 36.5, 47.9, 80.4, 120.0, 129.2, 132.5, 143.4, 144.4.

*6-bromo-3,4-dihydro-2H-spiro[naphthalene-1,3′-oxetane]* (**5b**): Yellow oil. 48% yield. HPLC: 8.230 min (98%). EI-MS: Calculated 252.01 for C_12_H_13_BrO. Found: 221.9 [M-CH_2_O]^+●^. ^1^H-NMR (200 MHz, CDCl_3_) δ in ppm: 1.65–1.77 (m, 2H), 2.14–2.20 (t, 2H), 2.69–2.75 (t, 2H), 4.62–4.65 (d, *J* = 6.0 Hz, 2H), 4.75–4.78 (d, *J* = 6.0 Hz, 2H), 7.21–7.22 (s, 1H), 7.38–7.43 (d, 1H), 7.77–7.81 (d, 1H). ^13^C-NMR (50 MHz, CDCl_3_) δ in ppm: 20.0, 29.9, 35.3, 42.2, 85.4, 120.4, 128.4, 129.9, 131.7, 138.7, 139.2.

*5-chloro-2,3-dihydrospiro[indene-1,3′-oxetane]* (**11**): Yellow oil. 47% yield. HPLC: 7.664 min (97%). EI-MS: Calculated 194.0 for C_11_H_11_ClO. Found: 163.9 [M-CH_2_O]^+●^. ^1^H-NMR (200 MHz, CDCl_3_) δ in ppm: 2.43–2.50 (t, *J* = 6.0 Hz, 2H), 2.83–2.90 (t, *J* = 6.0 Hz, 2H), 4.80 (s, 4H), 7.17 (s, 1H), 7.18–7.29 (d, 1H), 7.56–7.60 (d, 1H). ^13^C-NMR (50 MHz, CDCl_3_) δ in ppm: 30.4, 37.9, 50.9, 84.2, 123.8, 124.8, 127.4, 133.4, 144.7, 145.4.

*3-(4-bromophenyl)-3-methyloxetane* (**5c**): Yellow solid. 35% yield. HPLC: 7.390 min (98%). EI-MS: Calculated 226.00 for C_10_H_11_BrO. Found: 195.9 [M-CH_2_O]^+●^. ^1^H-NMR (200 MHz, CDCl_3_) δ in ppm: 1.71 (s, 3H), 4.62–4.64 (d, 2H), 4.90–4.93 (d, 2H), 7.07–7.11 (d, *J* = 8.0 Hz, 2H), 7.46–7.50 (d, *J* = 8.0 Hz, 2H). ^13^C-NMR (50 MHz, CDCl_3_) δ in ppm: 27.7, 43.3, 83.64, 120.3, 127.0, 131.8, 145.6.

### 3.9. General Procedure I—Oxetane Formation via Friedel-Crafts Reaction

Quantities of 0.008 g (0.028 mmol) of lithium bis(trifluoromethanesulfonimide) and 0.006 g (0.014 mmol) of tetrabutylammonium hexafluorophosphate were added to a solution of 0.066 g (0.258 mmol) of 3-(4-(benzyloxy)phenyl)oxetan-3-ol and 0.12 g (1.288 mmol) of phenol in 0.5 mL of chloroform. The reaction mixture was stirred at 40 °C for 1 h then quenched with sat. aq. NaHCO_3_. CH_2_Cl_2_ was added and the layers were separated. The organic extracts were combined, dried over Na_2_SO_4_, filtered and concentrated in vacuum. Purification by column chromatography (30% EtOAc:*n*-hexane) afforded the oxetane. Adapted from Croft et al. [28].

*4-(3-(4-(benzyloxy)phenyl)oxetan-3-yl)phenol* (**14**): White solid. 60% yield. HPLC: 8.286 min (93%). ESI-MS: Calculated: 332.14 for C_22_H_20_O_3_. Found: 331.1 [M − H]^−^. ^1^H-NMR (200 MHz, CDCl_3_) δ in ppm: 5.06 (s, 2H), 5.21 (s, 4H), 6.78–6.82 (d, *J* = 8.0 Hz, 2H), 6.94–6.98 (d, *J* = 8.0 Hz, 2H), 7.05–7.15 (m, 4H), 7.36–7.42 (m, 5H); ^13^C-NMR (50 MHz, CDCl_3_) δ in ppm: 50.5, 70.2, 85.2, 114.9, 115.5, 127.6, 127.8, 127.9, 128.2, 128.8, 137.1, 138.3, 138.5, 154.4, 157.6.

### 3.10. General Procedure J—Triflate Formation

First, 0.2 mL (2.708 mmol) of pyridine, then 0.3 mL (2.031 mmol) of triflic anhydride were added to a solution of 4-(3-(4-(benzyloxy)phenyl)oxetan-3-yl)phenol in 3.0 mL of CH_2_Cl_2_. The reaction mixture was stirred at room temperature. After controlling through TLC and HPLC, the work-up was done adding water followed by CH_2_Cl_2_. The layers were separated and the aqueous portion was extracted with CH_2_Cl_2_. The organic extracts were combined, dried over Na_2_SO_4_, filtered and concentrated in vacuum. Purification by column chromatography (20% EtOAc:*n*-hexane) afforded the triflate. Adapted from Thompson et al. [30].

*4-(3-(4-(benzyloxy)phenyl)oxetan-3-yl)phenyl trifluoromethanesulfonate* (**15**): White solid. 90% yield. HPLC: 10.332 min (100%). ESI-MS: Calculated: 464.09 for C_23_H_19_F_3_O_5_S. Found: 463.0 [M − H]^−^. ^1^H-NMR (200 MHz, CDCl_3_) δ in ppm: 5.06 (s, 2H), 5.21 (s, 4H), 6.78–6.82 (d, *J* = 8.0 Hz, 2H), 6.94–6.98 (d, *J* = 8.0 Hz, 2H),7.05–7.15 (m, 4H), 7.36–7.42 (m, 5H).

### 3.11. General Procedure K—Buchwald-Hartwig Reaction via Halides

A mixture of 1.0 eq. of aryl halide, 1.0 eq. of amine, 3.0 eq. of Cs_2_CO_3_ as the base, 0.5 eq. of X-Phos as the ligand and 0.1 eq. of Pd(OAc)_2_ as the catalyst in dry 1,4-dioxane and absolute tert-butanol (4:1) (0.05 M) was stirred at reflux under atmosphere of argon. After controlling through TLC and HPLC, the mixture was allowed to cool to room temperature. It was diluted with water and subsequently extracted with ethyl acetate. The extracts were combined, washed with brine and afterwards dried over Na_2_SO_4_. The solvent was removed under vacuum, and the residue was purified by column chromatography, affording the desired product. Adapted from Martz et al. [31].

*N-phenyl-6,7,8,9-tetrahydrospiro[benzo[7]annulene-5,3′-oxetan]-2-amine* (**16a**): Brown solid. 86% yield. HPLC: 9.015 min (96%). ESI-MS: Calculated: 279.1 for C_19_H_21_NO. Found: 278.0 [M − H]^−^. ^1^H-NMR (400 MHz, CDCl_3_) δ in ppm: 1.59 (m, 2H, CH_2_), 2.01 (m, 2H, CH_2_), 2.18 (t, 2H, *J* = 8.0 Hz, CH_2_), 2.42 (t, 2H, *J* = 8.0 Hz, CH_2_), 4.73 (d, 2H), 5.04 (d, 2H), 5.78 (br, 1H), 6.82 (s, 1H), 6.91–7.00 (m, 3H), 7.08 (d, 2H, *J* = 8.0 Hz),7.27 (t, 2H, *J* = 8.0 Hz); ^13^C-NMR (100 MHz, CDCl_3_) δ in ppm: 26.5, 27.3, 35.5, 37.1, 47.7, 80.7, 115.1, 117.9, 119.4, 121.0,126.2, 129.5, 137.1, 141.8, 143.4.

*N-(2,4-difluorophenyl)-6,7,8,9-tetrahydrospiro[benzo[7]annulene-5,3′-oxetan]-2-amine* (**16b**): Brown solid. 73% yield. HPLC: 8.542 min (98%). ESI-MS: Calculated: 315.1 for C_19_H_19_F_2_NO. Found: 314.2 [M − H]^−^. ^1^H-NMR (400 MHz, CDCl_3_) δ in ppm: 1.49 (m, 2H), 1.91 (m, 2H), 2.08 (t, 2H), 2.31 (t, 2H), 4.63–4.64 (d, 2H, *J* = 4.0 Hz), 4.93–4.94 (d, 2H, *J* = 4.0 Hz), 5.46 (br, 1H), 6.66 (s, 1H), 6.70–6.82 (m, 3H), 6.89 (d, 1H, *J* = 8.0 Hz), 7.18 (s, 1H); ^13^C-NMR (100 MHz, CDCl_3_) δ in ppm: 26.5, 27.3, 37.1, 47.7, 80.7, 104.1 (dd, *J*_1_ = 23.6 Hz, *J*_2_ = 23.7 Hz), 110.9 (dd, *J*_1_ = 3.64 Hz, *J*_2_ = 3.71 Hz), 114.9, 119.3, 119.5 (dd, *J*_1_ = 2.28 Hz, *J*_2_ = 3.17 Hz), 126.4, 127.9 (dd, *J*_1_ = 2.35 Hz, *J*_2_ = 2.36 Hz), 137.6, 141.4, 143.6.

*N-(2-(trifluoromethyl)phenyl)-6,7,8,9-tetrahydrospiro[benzo[7]annulene-5,3′-oxetan]-2-amine* (**16c**): Yellow solid. 77% yield. HPLC: 10.008 min (96%). ESI-MS: Calculated: 347.1 for C_20_H_20_F_3_NO. Found: 346.1 [M − H]^−^. ^1^H-NMR (400 MHz, CDCl_3_) δ in ppm: 1.58 (m, 2H), 1.99 (m, 2H), 2.17 (t, 2H), 2.42 (t, 2H), 4.73 (d, 2H), 5.02 (d, 2H), 6.02 (br, 1H), 6.84 (s, 1H), 6.90–6.96 (m, 2H), 7.01 (d, 1H, *J* = 8.0 Hz), 7.32–7.39 (m, 2H), 7.54 (d, 1H, *J* = 8.0 Hz); ^13^C-NMR (100 MHz, CDCl_3_) δ in ppm: 26.5, 27.2, 35.4, 36.9, 47.8, 80.7, 117.2, 117.5, 117.7 (d, *J* = 10.6 Hz), 119.6, 122.0, 123.6, 126.4, 127.0 (dd, *J*_1_ = 5.5 Hz, *J*_2_ = 5.4 Hz), 132.8, 139.0, 140.1, 142.5, 143.7.

*N-(4-methoxyphenyl)-6,7,8,9-tetrahydrospiro[benzo[7]annulene-5,3′-oxetan]-2-amine* (**16d**): Yellow solid. 86% yield. HPLC: 7.976 min (98%). ESI-MS: Calculated: 309.1 for C_20_H_23_NO_2_. Found: 308.0 [M − H]^−^. ^1^H-NMR (200 MHz, CDCl_3_) δ in ppm: 1.58 (m, 2H), 1.98 (m, 2H), 2.17 (t, 2H), 2.43 (t, 2H), 3.80 (s, 3H), 4.67 (d, 2H, *J* = 6.0 Hz), 4.99 (d, 2H, *J* = 6.0 Hz), 5.45 (br, 1H), 6.63–6.74 (m, 3H), 6.84–6.93 (m, 3H), 7.04 (t, 2H); ^13^C-NMR (100 MHz, CDCl_3_) δ in ppm: 26.6, 27.3, 35.6, 37.2, 47.9, 55.7, 112.8, 114.8, 117.3, 122.2, 126.2, 135.6, 136.0, 143.3, 143.9, 155.4.

*N-phenyl-3,4-dihydro-2H-spiro[naphthalene-1,3′-oxetan]-6-amine* (**17a**): Brown solid. 92% yield. HPLC: 7.967 min (97%). ESI-MS: Calculated: 265.1 for C_18_H_19_NO. Found: 264.0 [M − H]^−^. ^1^H-NMR (200 MHz, CDCl_3_) δ in ppm: 1.75–1.84 (m, 2H), 2.24 (t, 2H, *J* = 6.0 Hz), 2.75 (t, 2H, *J* = 6.0 Hz), 4.66 (d, 2H, *J* = 6.0 Hz), 4.87 (d, 2H, *J* = 6.0 Hz), 6.84 (s, 1H), 6.94–7.10 (m, 4H), 7.33 (t, 2H), 7.83 (d, 2H, *J* = 8.0 Hz); ^13^C-NMR (50 MHz, CDCl_3_) δ in ppm: 20.3, 30.4, 35.9, 42.1, 85.8, 116.8, 117.4, 117.9, 121.1, 127.7, 129.5, 132.1, 138.1, 141.7, 143.2.

*N-(2,4-difluorophenyl)-3,4-dihydro-2H-spiro[naphthalene-1,3′-oxetan]-6-amine* (**17b**): Brown solid. 51% yield. HPLC: 8.586 min (98%). ESI-MS: Calculated: 301.1 for C_18_H_17_F_2_NO. Found: 299.9 [M − H]^−^. ^1^H-NMR (200 MHz, CDCl_3_) δ in ppm: 1.70–1.79 (m, 2H), 2.19 (t, 2H, *J* = 6.0 Hz), 2.70 (t, 2H, *J* = 6.0 Hz), 4.61 (d, 2H, *J* = 6.0 Hz), 4.80 (d, 2H, *J* = 6.0 Hz), 6.70–6.99 (m, 4H), 7.19–7.31 (m, 1H), 7.79 (d, 2H, *J* = 10 Hz); ^13^C-NMR (50 MHz, CDCl_3_) δ in ppm: 20.3, 30.4, 35.8, 42.1, 85.8, 103.9 (dd, *J*_1_ = 23.5 Hz, *J*_2_ = 23.5 Hz), 110.8 (dd, *J*_1_ = 3.76 Hz, *J*_2_ = 3.79 Hz), 116.5, 117.3, 119.6 (dd, *J*_1_ = 3.22 Hz, *J*_2_ = 3.30 Hz), 127.9, 132.7, 138.3, 141.3, 151.0 (d, *J* = 11.8 Hz), 154.5 (d, *J* = 11.1 Hz), 155.9 (d, *J* = 11.8 Hz), 159.3 (d, *J* = 11.3 Hz).

*N-(2-(trifluoromethyl)phenyl)-3,4-dihydro-2H-spiro[naphthalene-1,3′-oxetan]-6-amine* (**17c**): White solid. 82% yield. HPLC: 9.943 min (98%). ESI-MS: Calculated: 333.1 for C_19_H_18_F_3_NO. Found: 332.2 [M − H]^−^. ^1^H-NMR (200 MHz, CDCl_3_) δ in ppm: 1.71–1.74 (m, 2H), 2.20 (t, 2H, *J* = 6.0 Hz), 2.72 (t, 2H, *J* = 6.0 Hz), 4.63 (d, 2H, *J* = 6.0 Hz), 4.82 (d, 2H, *J* = 6.0 Hz), 6.03 (br, 1H), 6.83 (s, 1H), 6.94 (t, 1H, *J* = 8.0 Hz), 7.06 (d, 1H, *J* = 8.0 Hz), 7.32–7.43 (m, 2H), 7.54 (d, 1H, *J* = 8.0 Hz), 7.84 (d, 1H, *J* = 8.0 Hz); ^13^C-NMR (50 MHz, CDCl_3_) δ in ppm: 20.3, 30.3, 35.8, 42.2, 85.8, 117.3, 117.9, 118.0, 118.9, 119.8, 120.1, 126.9 (dd, *J*_1_ = 5.5 Hz, *J*_2_ = 5.5 Hz), 127.9, 132.7, 134.1, 138.3, 140.1, 142.2.

*N-(2-methoxyphenyl)-3,4-dihydro-2H-spiro[naphthalene-1,3′-oxetan]-6-amine* (**17d**): Yellow solid. 82% yield. HPLC: 9.221 min (95%). ESI-MS: Calculated: 295.1 for C_19_H_21_NO_2_. Found: 294.4 [M − H]^−^. ^1^H-NMR (200 MHz, CDCl_3_) δ in ppm: 1.72–1.83 (m, 2H), 2.24 (t, 2H, *J* = 6.0 Hz), 2.76 (t, 2H, *J* = 6.0 Hz), 3.93 (s, 3H), 4.66 (d, 2H, *J* = 6.0 Hz), 4.87 (d, 2H, *J* = 6.0 Hz), 6.92 (m, 4H), 7.13 (d, 1H), 7.32 (m, 1H), 7.84 (d, 1H, *J* = 8.0 Hz); ^13^C-NMR (50 MHz, CDCl_3_) δ in ppm: 20.4, 30.4, 35.9, 42.1, 55.7, 85.8, 110.7, 114.9, 117.5, 118.2, 120.0, 120.9, 127.7, 132.3, 133.0, 138.0, 141.3, 148.4.

*N-phenyl-2,3-dihydrospiro[indene-1,3′-oxetan]-5-amine* (**18a**): Brown solid. 71% yield. HPLC: 8.231 min (95%). ESI-MS: Calculated: 251.1 for C_17_H_17_NO. Found: 250.1 [M − H]^−^. ^1^H-NMR (200 MHz, CDCl_3_) δ in ppm: 2.52 (t, 2H, *J* = 8.0 Hz), 2.89 (d, 2H, *J* = 8.0 Hz), 4.83–4.92 (m, 4H), 5.82 (br, 1H), 6.94–7.12 (m, 5H), 7.28–7.36 (m, 2H), 7.57 (d, 2H, *J* = 8.0 Hz); ^13^C-NMR (50 MHz, CDCl_3_) δ in ppm: 30.6, 38.4, 50.9, 84.6, 113.8, 117.4, 177.9, 121.1, 123.6, 129.5, 138.8, 143.0, 143.5, 144.9.

*N-(2,4-difluorophenyl)-2,3-dihydrospiro[indene-1,3′-oxetan]-5-amine* (**18b**): Brown solid. 76% yield. HPLC: 8.670 min (100%). ESI-MS: Calculated: 287.1 for C_17_H_15_F_2_NO. Found: 286.1 [M − H]^−^. ^1^H-NMR (200 MHz, CDCl_3_) δ in ppm: 2.47 (t, 2H, *J* = 6.0 Hz), 2.89 (d, 2H, *J* = 6.0 Hz), 4.79–4.87 (m, 4H), 6.76–6.98 (m, 4H), 7.17–7.29 (m, 1H), 7.53 (d, 2H, *J* = 8.0 Hz); ^13^C-NMR (100 MHz, CDCl_3_) δ in ppm: 30.6, 38.3, 50.9, 84.6, 103.9, 104.4 (*J* = 2.87 Hz), 110.8 (*J*_1_ = 3.83, *J*_2_ = 3.97 Hz), 113.6, 117.3, 119.6 (*J*_1_ = 2.86, *J*_2_ = 3.34 Hz), 123.7, 139.3, 142.7, 145.1.

*N-(2-(trifluoromethyl)phenyl)-2,3-dihydrospiro[indene-1,3′-oxetan]-5-amine* (**18c**): White solid. 95% yield. HPLC: 9.588 min (100%). ESI-MS: Calculated: 319.1 for C_18_H_16_F_3_NO. Found: 318.1 [M − H]^−^. ^1^H-NMR (200 MHz, CDCl_3_) δ in ppm: 2.49 (t, 2H, *J* = 8.0 Hz), 2.86 (d, 2H, *J* = 8.0 Hz), 4.80–4.88 (m, 4H), 6.06 (br, 1H), 6.90–6.97 (m, 2H), 7.04 (d, 2H, *J* = 8.0 Hz), 7.29–7.41 (m, 2H), 7.58 (t, 2H, *J* = 8.0 Hz); ^13^C-NMR (50 MHz, CDCl_3_) δ in ppm: 30.6, 38.2, 50.9, 84.5, 116.5, 117.9, 119.8, 123.7, 127.1, 132.8, 140.7, 141.4, 142.5, 145.1.

*N-(2-methoxyphenyl)-2,3-dihydrospiro[indene-1,3′-oxetan]-5-amine* (**18d**): Yellow solid. 80% yield. HPLC: 8.718 min (98%). ESI-MS: Calculated: 281.1 for C_18_H_19_NO_2_. Found: 280.1 [M − H]^−^. ^1^H-NMR (200 MHz, CDCl_3_) δ in ppm: 2.48 (t, 2H, *J* = 8.0 Hz), 2.86 (d, 2H, *J* = 8.0 Hz), 3.89 (s, 3H), 4.79–4.89 (m, 4H), 6.15 (br, 1H), 6.85–6.91 (m, 3H), 7.02 (s, 1H), 7.07 (d, 1H), 7.28 (m, 1H), 7.54 (d, 2H, *J* = 8.0 Hz); ^13^C-NMR (50 MHz, CDCl_3_) δ in ppm: 30.6, 38.4, 50.9, 55.7, 84.6, 110.6, 114.5, 114.8, 118.1, 119.9, 120.9, 123.5, 133.3, 138.9, 142.6, 144.8, 148.3.

*4-(3-methyloxetan-3-yl)-N-phenylaniline* (**19a**): Brown solid. 83% yield. HPLC: 7.967 min (96%). ESI-MS: Calculated: 239.1 for C_16_H_17_NO. Found: 238.0 [M − H]^−^. ^1^H-NMR (400 MHz, CDCl_3_) δ in ppm: 1.75 (s, 3H, CH_3_), 4.65–4.66 (d, 2H, *J* = 4.0 Hz, CH_2_), 4.98–4.99 (d, 2H, *J* = 4.0 Hz, CH_2_), 5.76 (br, 1H), 6.94–6.97 (t, 1H, *J* = 8.0 Hz), 7.08–7.11 (m, 4H), 7.16–7.18 (d, 2H, *J* = 8.0 Hz), 7.28–7.36 (t, 2H, *J* = 8.0 Hz); ^13^C-NMR (100 MHz, CDCl_3_) δ in ppm: 27.7, 43.0, 84.2, 117.8, 118.2, 121.1, 126.2, 129.5, 139.1, 141.6, 143.4.

*2,4-difluoro-N-(4-(3-methyloxetan-3-yl)phenyl)aniline* (**19b**): Brown solid. 71% yield. HPLC: 7.689 min (100%). ESI-MS: Calculated: 275.1 for C_16_H_15_F_2_NO. Found: 274.0 [M − H]^−^. ^1^H-NMR (200 MHz, CDCl_3_) δ in ppm: 1.73 (s, 3H), 4.62 (d, 2H, *J* = 6.0 Hz), 4.94 (d, 2H, *J* = 6.0 Hz), 6.75–6.95 (m, 2H), 6.99 (d, 2H, *J* = 10 Hz), 7.14 (d, 2H, *J* = 8.0 Hz), 7.23–7.28 (d, 1H); ^13^C-NMR (50 MHz, CDCl_3_) δ in ppm: 27.7, 42.9, 84.1, 103.9 (dd, *J*_1_ = 23.5 Hz, *J*_2_ = 23.5 Hz), 110.8 (dd, *J*_1_ = 3.8 Hz, *J*_2_ = 3.8 Hz), 117.9, 119.3 (dd, *J*_1_ = 3.4 Hz, *J*_2_ = 3.3 Hz), 126.3, 127.8 (dd, *J*_1_ = 3.4 Hz, *J*_2_ = 3.1 Hz), 139.6, 141.2, 150.9 (d, *J* = 11.9 Hz), 154.5 (d, *J* = 11.1 Hz), 155.8 (d, *J* = 11.7 Hz), 159.3 (d, *J* = 11.1 Hz).

*N*-(4-(3-methyloxetan-3-yl)phenyl)-2-(trifluoromethyl)aniline (**19c**): White solid. 88% yield. HPLC: 9.306 min (100%). ESI-MS: Calculated: 307.1 for C_17_H_16_F_3_NO. Found: 306.0 [M − H]^−^. ^1^H-NMR (400 MHz, CDCl_3_) δ in ppm: 1.78 (s, 3H, CH_3_), 4.67–4.70 (d, 2H, *J* = 6.0 Hz, CH_2_), 4.99–5.02 (d, 2H, *J* = 6.0 Hz, CH_2_), 6.10 (br, 1H), 6.94–7.02 (t, 1H, *J* = 8.0 Hz), 7.13–7.25 (q, 4H), 7.30–7.41 (m, 2H), 7.59–7.63 (d, 2H, *J* = 8.0 Hz); ^13^C-NMR (100 MHz, CDCl_3_) δ in ppm: 27.7, 43.1, 84.0, 114.3, 117.7, 119.8, 120.7, 126.4, 127.1, 132.8, 140.0, 140.7, 141.1, 142.4.

*2-methoxy-N-(4-(3-methyloxetan-3-yl)phenyl)aniline* (**19d**): Yellow solid. 81% yield. HPLC: 8.251 min (98%). EI-MS: Calculated: 269.1 for C_17_H_19_NO_2_. Found: 269.1. ^1^H-NMR (400 MHz, CDCl_3_) δ in ppm: 1.73 (s, 3H, CH_3_), 3.90 (s, 3H, OCH_3_), 4.62–4.64 (d, 2H, *J* = 8.0 Hz, CH_2_), 4.96–4.97 (d, 2H, *J* = 4.0 Hz, CH_2_), 6.85–6.90 (m, 3H), 7.15 (s, 4H), 7.27–7.29 (d, 1H); ^13^C-NMR (100 MHz, CDCl_3_) δ in ppm: 27.7, 43.0, 55.7, 84.2, 110.7, 114.7, 118.9, 120.0, 120.9, 126.1, 133.2, 139.2, 141.2, 148.4.

### 3.12. General Procedure L—Buchwald–Hartwig Reaction via Triflates—Benzyl Ether Deprotection

A mixture of aryl triflate, amine, Cs_2_CO_3_ as the base, BINAP as the ligand and Pd(OAc)_2_ as the catalyst in dry 1,4-dioxane and absolute tert-butanol (4:1) (0.03 M) was stirred at 110 °C under an atmosphere of argon. After controlling through TLC and HPLC, the mixture was allowed to cool to room temperature. It was diluted with water and subsequently extracted with ethyl acetate. The extracts were combined, washed with brine and afterwards dried over Na_2_SO_4_. The solvent was removed under vacuum, and the residue was purified by column chromatography, affording the product. Adapted from Åhman and Buchwald [33].

Then the benzyl ether deprotection was performed as follows: 0.6 eq. of palladium on carbon (10% *w/w*) was added to a solution of oxetane in ethanol/acetonitrile. The reaction mixture was stirred under an atmosphere of hydrogen at 25 °C until HPLC analysis indicated the end of reaction. The reaction mixture was filtered through celite, washed through with ethanol and then concentrated in vacuum to afford the final oxetane. Adapted from Croft et al. [28].

*4-(3-(4-(phenylamino)phenyl)oxetan-3-yl)phenol* (**20a**): White solid. 85% yield. HPLC: 7.618 min (96%). ESI-MS: Calculated: 317.1 for C_21_H_19_NO_2_. Found: 316.2 [M − H]^−^. ^1^H-NMR (400 MHz, *d*-acetone) δ in ppm: 5.13 (s, 4H), 6.82–6.85 (t, 3H), 7.10–7.17 (m, 8H), 7.20–7.24 (t, 2H), 7.41 (br, 1H); ^13^C-NMR (100 MHz, *d*-acetone) δ in ppm: 51.3, 84.9, 116.1, 117.9, 118.1, 120.9, 128.0, 129.9, 138.5, 139.5, 142.9, 144.7, 156.7.

*4-(3-(4-((2,4-difluorophenyl)amino)phenyl)oxetan-3-yl)phenol* (**20b**): Brown solid. 66% yield. HPLC: 7.930 min (98%). ESI-MS: Calculated: 353.1 for C_21_H_17_F_2_NO_2_. Found: 352.0 [M − H]^−^. ^1^H-NMR (400 MHz, *d*-acetone) δ in ppm: 5.12 (s, 4H), 6.81–6.83 (d, 2H, *J* = 8.0 Hz), 6.90–6.95 (t, 1H), 7.0–7.02 (d, 2H, *J* = 8.0 Hz), 7.11–7.13 (d, 2H, *J* = 8.0 Hz), 7.15–7.17 (d, 2H, *J* = 8.0 Hz), 7.32–7.38 (m, 1H), 8.29 (br, 1H); ^13^C-NMR (100 MHz, *d*-acetone) δ in ppm: 51.6, 85.0, 104.9, 105.1 (d, *J* = 3.0 Hz), 105.4, 111.9 (d, *J* = 4.0 Hz), 112.1 (d, *J* = 4.0 Hz), 116.3, 117.9, 122.1 (d, *J* = 3.0 Hz), 122.2 (d, *J* = 3.0 Hz), 128.3, 128.5, 138.7, 140.0, 143.3, 156.9.

*4-(3-(4-((4-(trifluoromethyl)phenyl)amino)phenyl)oxetan-3-yl)phenol* (**20c**): White solid. 71% yield. HPLC: 7.967 min (96%). ESI-MS: Calculated: 385.1 for C_22_H_18_F_3_NO_2_. Found: 383.9 [M − H]^−^. ^1^H-NMR (200 MHz, *d*-acetone) δ in ppm: 5.15 (s, 4H), 6.82–6.86 (d, 2H, *J* = 8.0 Hz), 7.11–7.29 (m, 8H), 7.49–7.53 (d, 2H, *J* = 8.0 Hz), 7.93 (br, 1H), 8.34 (br, 1H); ^13^C-NMR (50 MHz, *d*-acetone) δ in ppm: 51.5, 84.8, 115.7, 116.1, 120.5, 121.0, 127.3, 127.4, 128.2, 128.4, 138.3, 140.9, 141.8, 148.8, 156.8.

*4-(3-(4-((4-methoxyphenyl)amino)phenyl)oxetan-3-yl)phenol* (**20d**): Yellow solid. 54% yield. HPLC: 7.159 min (99%). ESI-MS: Calculated: 347.1 for C_22_H_21_NO_3_. Found: 345.9 [M − H]^−^. ^1^H-NMR (400 MHz, *d*-acetone) δ in ppm: 3.75 (s, 3H), 5.11 (s, 4H), 6.80–6.89 (t, 4H), 6.93–6.97 (d, 2H, *J* = 8.0 Hz), 7.07–7.13 (m, 7H); ^13^C-NMR (100 MHz, *d*-acetone) δ in ppm: 27.7, 43.0, 84.2, 117.8, 118.2, 121.1, 126.2, 129.5, 139.1, 141.6, 143.4.

*4-((4-(3-(4-hydroxyphenyl)oxetan-3-yl)phenyl)amino)benzonitrile* (**20e**): Yellow solid. 70% yield. HPLC: 6.883 min (96%). ESI-MS: Calculated: 342.1 for C_22_H_18_N_2_O_2_. Found: 341.1 [M − H]^−^. ^1^H-NMR (200 MHz, *d*-acetone) δ in ppm: 5.15 (s, 4H), 6.81–6.86 (d, 2H, *J* = 10 Hz), 7.11–7.16 (d, 2H, *J* = 10 Hz), 7.20–7.31 (dd, 4H, *J* = 10 Hz, *J* = 8.0 Hz), 7.52–7.56 (d, 2H, *J* = 8.0 Hz), 8.12 (br, 1H), 8.32 (br, 1H); ^13^C-NMR (50 MHz, *d*-acetone) δ in ppm: 51.6, 84.8, 101.5, 115.7, 116.2, 120.4, 121.5, 128.4, 128.5, 134.5, 138.3, 140.1, 142.7, 149.7, 156.9.

## 4. Conclusions

We successfully synthesized the spiro and the methyl-phenyl oxetane scaffolds in good and reproducible yields. The hydroxyl replacement via oxyphosphonium salts proved to be a winning strategy for the synthesis of oxetanes from the aryl-alkyl diols through a cyclization reaction. Additionally, the reaction of phenol with a functionalized oxetan-3-ol was pivotal to obtain the 3,3-diaryloxetanes. Five scaffolds and novel compounds possessing different electronic properties were synthesized using the Buchwald–Hartwig reaction, which worked well for all halides and triflate derivatives.

We succeeded in finding three new hits against the CAMK1G and AAK1 through a screening in a kinase panel using the DSF method. The results indicate that it is possible to explore compounds bearing the oxetane motif in medicinal chemistry, aiming at the discovery of novel kinase inhibitor prototypes. However, some results demonstrated that the oxetane group is probably not interacting in the hinge region, which could be associated to conformational variations or to directional hydrogen bonding differences between sp_2_- and sp_3_-hybridized oxygen atoms.

As a perspective, new experiments could be carried out to determine the K_d_ and IC_50_ of the hit compounds. Furthermore, it is also possible to extensively expand the chemical space of oxetanes using other palladium-catalyzed coupling reactions, such as Sonogashira, Suzuki and Heck.

In addition to these preliminary results, new structural modifications and experiments to measure the K_d_ and IC_50_ will be carried out for a better understanding of the structure-activity relationship.

## Figures and Tables

**Table 1:** *N*-arylamine oxetanes obtained via Buchwald–Hartwig Reaction.

Entry	Halide/Triflate	Product		Yield (%) ^a^
**1**	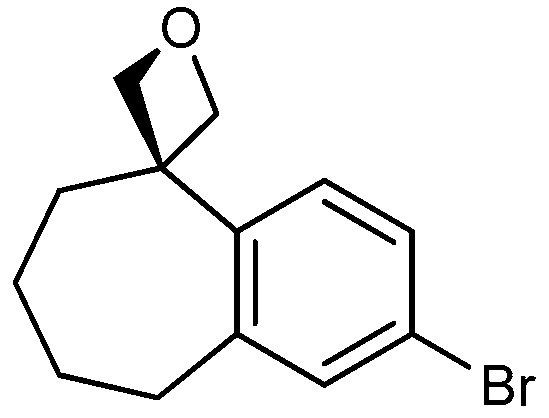	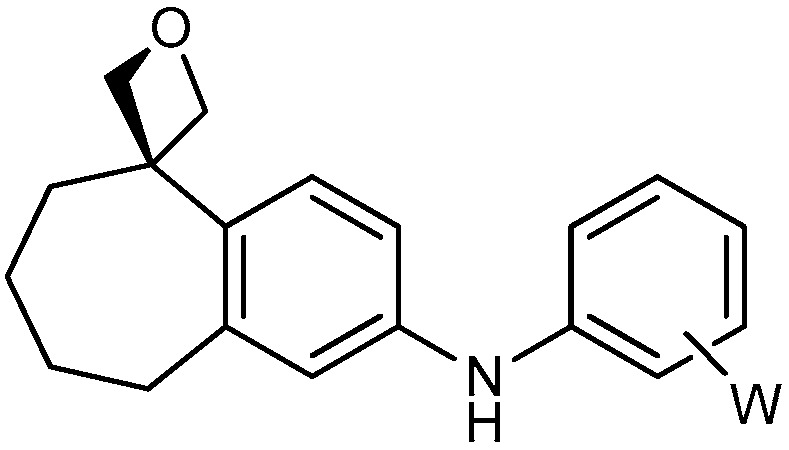	16a: W = H 16b: W = 2,4-di-F 16c: W = 2-CF_3_ 16d: W = 4-OCH_3_	86 73 77 86
**2**	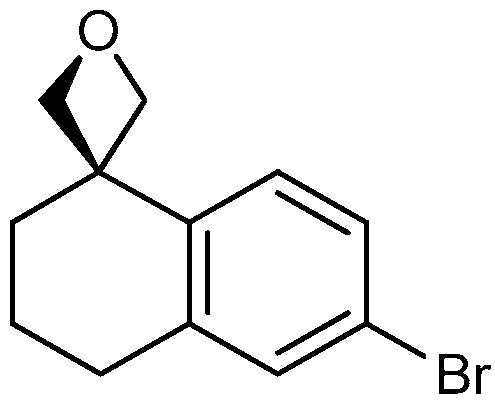	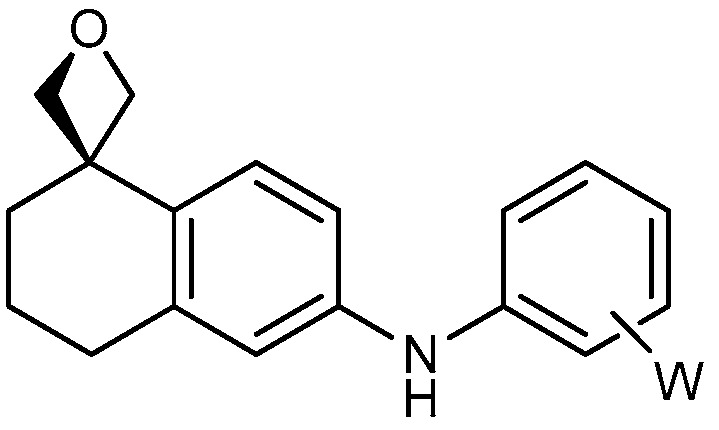	17a: W = H 17b: W = 2,4-di-F 17c: W = 2-CF_3_ 17d: W = 2-OCH_3_	92 51 82 82
**3**	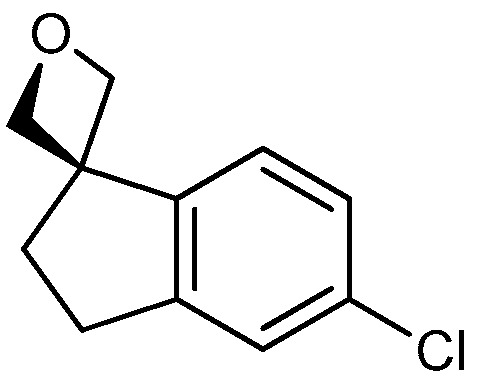	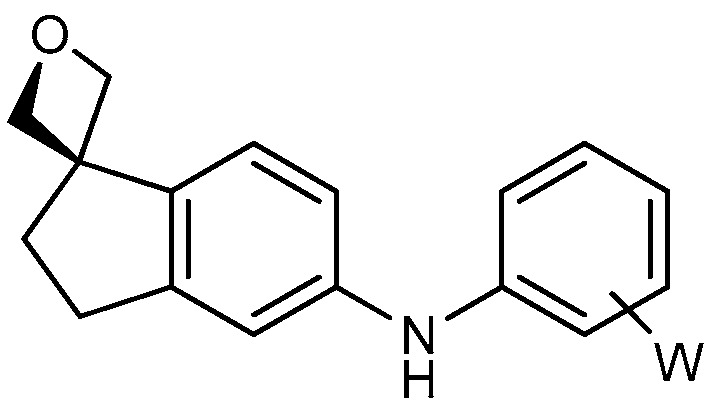	18a: W = H 18b: W = 2,4-di-F 18c: W = 2-CF_3_ 18d: W = 2-OCH_3_	71 76 95 80
**4**	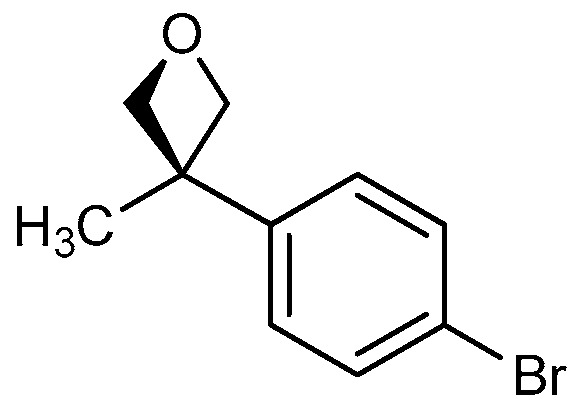	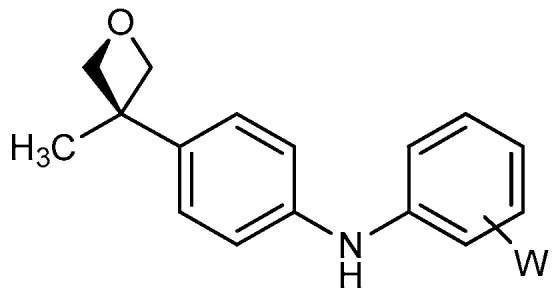	19a: W = H 19b: W = 2,4-di-F 19c: W = 2-CF_3_ 19d: W = 2-OCH_3_	83 71 88 81
**5 ^b^**	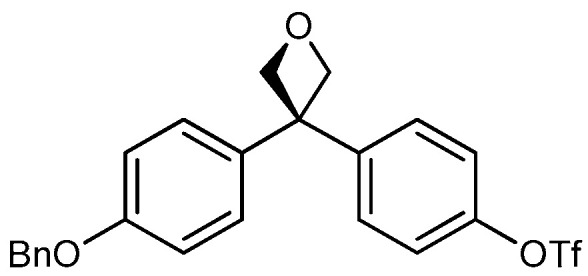	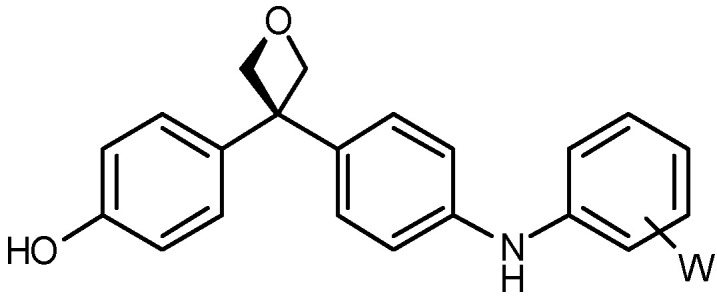	20a: W = H 20b: W = 2,4-di-F 20c: W = 4-CF_3_ 20d: W = 4-OCH_3_ 20e: W = 4-CN	85 66 71 54 70

^a^ Isolated yields after purification by column chromatography. ^b^ Products obtained after deprotection of benzyl ether group.

## Abbreviations

TDAP	Tris(dimethylamino)phosphine
ATP	Adenosine triphosphate
BINAP	2,2′-bis(diphenylphosphino)-1,1′-binaphthyl
Bu_4_NPF_6_	Tetrabutylammonium hexafluorophosphate
(CH_3_)_3_SI	Trimethylsulfonium iodide
Li(NTf_2_)	Lithium bis(trifluoromethanesulfonimide)
Ph_3_PMeI	Methyltriphenylphosphonium iodide
XPhos	2-Dicyclohexylphosphino-2′,4′,6′-triisopropylbiphenyl
